# Bayesian variable selection in modelling geographical heterogeneity in malaria transmission from sparse data: an application to Nouna Health and Demographic Surveillance System (HDSS) data, Burkina Faso

**DOI:** 10.1186/s13071-015-0679-7

**Published:** 2015-02-22

**Authors:** Eric Diboulo, Ali Sié, Diallo A Diadier, Dimitrios A Karagiannis Voules, Yazoume Yé, Penelope Vounatsou

**Affiliations:** Swiss Tropical and Public Health Institute, Basel, Switzerland; University of Basel, Basel, Switzerland; Centre de Recherche en Santé de Nouna, Nouna, Burkina Faso; PATH Malaria Vaccine Initiatives, Dakar, Senegal; ICF International, Rockville, MD 20850 USA

**Keywords:** Bayesian, Zero-inflated, Negative binomial, Stochastic search variable selection, Entomological inoculation rate, Burkina Faso, Sub-Saharan Africa, Nouna HDSS, Lag time

## Abstract

**Background:**

Quantification of malaria heterogeneity is very challenging, partly because of the underlying characteristics of mosquitoes and also because malaria is an environmentally driven disease. Furthermore, in order to assess the spatial and seasonal variability in malaria transmission, vector data need to be collected repeatedly over time (at fixed geographical locations). Measurements collected at locations close to each other and over time tend to be correlated because of common exposures such as environmental or climatic conditions. Non- spatial statistical methods, when applied to analyze such data, may lead to biased estimates. We developed rigorous methods for analyzing sparse and spatially correlated data. We applied Bayesian variable selection to identify the most important predictors as well as the elapsing time between climate suitability and changes in entomological indices.

**Methods:**

Bayesian geostatistical zero-inflated binomial and negative binomial models including harmonic seasonal terms, temporal trends and climatic remotely sensed proxies were applied to assess spatio-temporal variation of sporozoite rate and mosquito density in the study area. Bayesian variable selection was employed to determine the most important climatic predictors and elapsing (lag) time between climatic suitability and malaria transmission. Bayesian kriging was used to predict mosquito density and sporozoite rate at unsampled locations. These estimates were converted to covariate and season-adjusted maps of entomological inoculation rates. Models were fitted using Markov chain Monte Carlo simulation.

**Results:**

The results show that *Anophele. gambiae* is the most predominant vector (79.29%) and is more rain-dependant than its sibling *Anophele. funestus* (20.71%). Variable selection suggests that the two species react differently to different climatic conditions. Prediction maps of entomological inoculation rate (EIR) depict a strong spatial and temporal heterogeneity in malaria transmission risk despite the relatively small geographical extend of the study area.

**Conclusion:**

Malaria transmission is very heterogeneous over the study area. The EIR maps clearly depict a strong spatial and temporal heterogeneity despite the relatively small geographical extend of the study area. Model based estimates of transmission can be used to identify high transmission areas in order to prioritise interventions and support research in malaria epidemiology.

**Electronic supplementary material:**

The online version of this article (doi:10.1186/s13071-015-0679-7) contains supplementary material, which is available to authorized users.

## Background

Malaria is endemic in the majority of sub-Saharan Africa. It is transmitted from human to human via bites of mosquitoes infected with malaria parasites. A favorable environment and a complex system of malaria vectors and parasites maintain this endemicity. The mosquito development and survival strongly depend on prevailing climatic and environmental factors which in turn influence malaria transmission [1]. The species of vectors and their densities, the species of the malaria parasites, the number of infected bites a human received per night (a parameter known as the entomological inoculation rate, EIR) can change from place to place and according to the season. Therefore, malaria distribution is very heterogeneous within a geographical area and prone to between and within village variation [2,3].

There are two main species of malaria vector, *Anopheles gambiae* and *Anopheles funestus.* They differ in among others things, the type of water bodies in which they lay their eggs, their propensity to bite humans, the length of time for which they survive, the place where they rest after feeding, and time of the day when they bite. *An. gambiae*, the most efficient malaria vector, breeds in rice fields, sunlit pools both natural and man-made, and puddles. It is mainly endophilic (rests indooors) and also endophagic (feeds indoors) and favors pools produced by rainfall. *An. funestus* prefers shaded habitats and breeds in permanent waters, especially with vegetation. It bites humans and domestic animals and is both endophilic and exophilic [4]. Understanding the vector species' behavior and their interrelation with the environment is of prime importance in order to develop timely and effective intervention programs.

There is an elapsing time between climatic suitability, abundance of mosquito densities and onset of transmission. Changes in entomological parameters such as EIR depend on lag times and therefore it is important to take lag time into account in order to deliver timely and tailored interventions. A number of studies have used remote sensing (RS) climatic and environment proxies together to identify species-specific climatic predictors however few rigorously incorporate lag times into the analysis. RS data are often summarized by a long term average over a period of time prior to entomological data collection which is often considered as fixed rather than estimated by the data [5,6].

Estimating the lag times is not only important for delivering interventions but also for obtaining good predictive models to assess the distribution of mosquitos’ density. Entomological data are sparse and clustered in space and time due to spatial clustering of the environmental exposures and seasonality in transmission. Often the data include a large number of zeros (i.e. mosquito presence and/or infected) especially during the dry season. Zeros with frequencies higher than those expected by the data distribution (for counts or proportions) often lead to overdispersion and poor fit if they are not taken into account. Zero-Inflated (ZI) models provide a flexible way to address this problem [7] by assuming that only a proportion of the zeros arise from the data distribution and the remaining ones are “structural” (i.e. they appear with probability one).

Bayesian geostatistical models have been used to take into account spatio-temporal variation and zero-inflation in entomological data [5,6]; however lag times in climatic factors have not been rigorously incorporated into the modelling. Furthermore, selection of the climatic predictors to be included in the model is based on standard variable selection methods, which ignore spatio-temporal correlation. Recently, Bayesian variable selection methods have been used in modeling geostatistical survey data to identify the most important predictors of disease risk [8,9], however these methods have not been applied in modelling entomological data.

In this study, we apply zero-inflated models and introduce Bayesian variable selection to determine the elapsing time between climate suitability and malaria transmission and develop predictive models of EIR taking into account spatio-temporal heterogeneity and seasonality in terms of mosquito density and infectivity (sporozoite rate). We also determine the most important climatic predictors associated with the occurrence of the most predominant malaria vector species and transmission using data from the Nouna district in Burkina Faso.

## Methods

The data that motivated the present work were collected at Nouna Health and Demographic Surveillance System as part of the International Network for the Demographic Evaluation of Populations and Their Health-Malaria Transmission Intensity and Mortality Burden across Africa (INDEPTH-MTIMBA) protocol. INDEPTH-MTIMBA was a multi-centre project during 2001-2004 aimed at studying the relationship between the intensity of malaria transmission and all-cause as well as malaria specific mortality taking into account the influence of malaria control activities in each participant site. The Nouna HDSS is run by the Centre de recherche en santé de Nouna (CRSN, Nouna Health Research Center) and located in the Nouna health district’s catchment area in northwest Burkina Faso, 300 km away from the capital city, Ouagadougou. Relative to the health district, the HDSS catchment area is located southeast.

The Nouna HDSS area is characterized by a Sub-Saharan climate with a mean annual rainfall of approximately 800 mm with fairly constant average daily minimum (20-28.1°C) and maximum temperature (29.5-37.2°C) throughout the year. Rainfall occurs from May to September. The entire region consists of “Plateaux” with gentle slopes and drained by several small semi-permanent streams.

The HDSS area is about 1,775 km^2^ with the specificity of covering both rural and semi-urban areas.

The population is about 90,000 residing in 11,750 households across 58 villages and Nouna town. Subsistence farming is the predominant occupation. Malaria is holo-endemic and is known for a seasonal recrudescence during the rainy season, at which time it accounts for the main cause of fever and mortality in the district [10]. During the dry season, in February and March, lower respiratory infections are the main cause of morbidity, due to the relatively cool temperatures and strong winds, which bring up dust and dirt.

### Entomological data

Entomological data were collected using the Center for Disease Control and Prevention (CDC) light traps from 10 randomly selected compounds (from the HDSS database) over two consecutive nights every two weeks during the study period (September 2001- December 2003). In each house, a light trap was hung at about 1.5 m above the floor next to the bed of an index person and mosquitoes were collected for two consecutive nights. The sleeping place was covered with a bed net to protect the index person from mosquito bites. Other people in the same room without bed nets were also provided with untreated nets for these specific nights. Light traps were operated from dawn to dusk. All *Anopheles* mosquitoes captured were identified morphologically [11], stored and dried in vials with silica gel until they could be transported to the laboratory for further testing. The head and thorax of each anopheline was tested singly for *P. falciparum* sporozoites using a standard enzyme-linked immunosorbent assay (ELISA) [11]. To assess the seasonal pattern, data were summarized by location and calendar month. This implied that all surveys data collected within the same calendar month from a specific location (compound) were collapsed (mosquito density/tested and positive) into a single observation resulting in 160 and 285 unique locations respectively for sporozoite data of *An. funestus* and *An. gambiae* and 550 unique locations for density data for both species.

### Environmental and climatic data

Remote sensing data were used as proxies of climatic and environmental conditions. The predictors used, sources extracted and their spatio-temporal resolution are given in Table 1.

**Table 1:** **Sources of environmental and climatic predictors**

**Source**	**Predictor**	**Period**	**Spatial resolution**	**Temporal resolution**
Moderate Resolution Imaging Spectroradiometer (MODIS) Terra	Day & Night Land Surface Temperature (LST)	2001-2003	1 × 1 km^2^	8 days
Moderate Resolution Imaging Spectroradiometer (MODIS) Terra	Normalized Difference Vegetation Index (NDVI)	2001-2003	0.25 × 0.25 km^2^	16 days
Africa Data Disseminating Services	Rainfall	2001-2003	8 × 8 km^2^	10 days
Health Mapper	Water Bodies (Permanent & semi-permanent)	-	1 × 1 km^2^	na

To account for the environment-lagged effects on changes in mosquito density and infectivity, environmental factors were extracted up to three months prior to the month of mosquito collection for each surveyed location. Based on the biological plausibility (latent periods in the mosquito and parasite life cycle), six lag variables were constructed for each environmental factor (i.e. normalized difference vegetation index (NDVI), day land surface temperature (LST), night LST and rainfall) by averaging its values over the following periods: current month of the mosquito collection (Lag0), one and two month(s) prior to collection (Lag1, Lag2, respectively), average during the current and one previous month (Lag3), average during three months prior to the current one (Lag4) and average during the current and the two previous months (Lag5).

### Description of methods

We followed the approach by [5] and developed zero-inflated binomial (ZIB) and zero-inflated negative binomial (ZINB) models to model sporozoite rate (proportion of infected mosquitoes) and mosquito densities, respectively. We extended the methodology by introducing Bayesian variable selection to identify the most important climatic factors related to malaria transmission and take into account lag times between climatic suitability and malaria transmission. Four models were fitted separately to *An. funestus* and *An. gambiae* data to obtain species-specific surfaces of mosquito density and sporozoite rate within the study area. The overall EIR estimate at a given location and month are based on the mean number of infected mosquitoes (from both species) multiplied by the conversion factor. Modeling details are given bellow.

### Modeling sporozoite rate using zero-inflated binomial (ZIB)

Let ${N}_{it},{Y}_{it}^{(1)}$ be the number of tested and number of positive mosquitoes, respectively at location *i*, *i* = 1, … *n* and month *t* and ${\underset{\sim }{X}}_{it}$ is the set of predictors. We consider that ${Y}_{it}^{(1)}$ arises from a ZIB distribution, that is ${Y}_{it}^{(1)}\sim ZIB\left({N}_{it},{p}_{it},{\pi}_{it}^{(1)}\right)$ where *p*_*it*_ is the proportion of infected mosquitoes known as the sporozoite rate. Malaria seasonality introduces a large number of zero infected mosquitoes. The ZIB distribution assumes that a proportion ${\pi}_{it}^{(1)}$ (i.e. mixing proportion) of those zeros are “structural” (not random) and the remaining ones are present in the data with the frequency defined by the binomial distribution [7]. We model the relation between the sporozoite rate *p*_*it*_ and the environmental predictors via the logistic regression equation, $\log it\left({p}_{it}\right)={{\underset{\sim }{X}}^T}_{it}{\underset{\sim }{\beta}}^{(1)}$ where ${\underset{\sim }{\beta}}^{(1)}$ is the set of regression coefficients. We also assume that the mixing proportion of zeros is also influenced by climatic factors ${{\underset{\sim }{Z}}^T}_{it}$ which we introduce into the modelling by the equation $\log it\left({\pi}_{it}^{(1)}\right)={{\underset{\sim }{Z}}^T}_{it}{\underset{\sim }{\gamma}}^{(1)}$, where ${\underset{\sim }{\gamma}}^{(1)}$ is the set of corresponding coefficients.

### Modeling mosquito densities using zero-inflated negative binomial (ZINB)

Let ${Y}_{it}^{(2)}$ be the number of mosquitoes trapped at location *i* and time *t*. We assume that ${Y}_{it}^{(2)}$ arises from a ZINB distribution, ${Y}_{it}^{(2)}\sim ZINB\left({\mu}_{it},r,{\pi}_{it}^{(2)}\right)$ with *μ*_*it*_ and *r* corresponding to the mean mosquito count and variance of the negative binomial distribution [12]. ${\pi}_{it}^{(2)}$ corresponds to the mixing proportion modelling the “excess zeros”. As defined above it is considered that a proportion of the zero mosquito counts is “structural” and the remaining $1-{\pi}_{it}^{(2)}$ arise from the negative binomial distribution. We model the relation between the mean mosquito density *μ*_*it*_, mixing proportion of zeros ${\pi}_{it}^{(2)}$ and climatic predictors by the equations $\log \left({\mu}_{it}\right)={{\underset{\sim }{X}}^T}_{it}{\underset{\sim }{\beta}}^{(2)}$ and $\log it\left({\pi}_{it}^{(2)}\right)={{\underset{\sim }{Z}}^T}_{it}{\underset{\sim }{\gamma}}^{(2)}$ where ${\underset{\sim }{\beta}}^{(2)}$ and ${\underset{\sim }{\gamma}}^{(2)}$ the regression coefficients.

### Modeling spatio-temporal heterogeneity

We extend the above formulation to include seasonality, spatial and temporal correlation on the sporozoite rates as well as the mosquito density, that is, $\log it\left({p}_{it}\right)={{\underset{\sim }{X}}^T}_{it}{\underset{\sim }{\beta}}^{(1)}+{f}_{(1)}(t)+{\phi_i}^{(1)}+{\varepsilon}_t^{(1)}$ and $\log \left({\mu}_{it}\right)={{\underset{\sim }{X}}^T}_{it}{\underset{\sim }{\beta}}^{(2)}+{f}_{(2)}(t)+{\phi_i}^{(2)}+{\varepsilon}_t^{(2)}$, where *f*_(*k*)_(*t*) captures seasonal patterns, ${\phi_i}^{(k)},{\varepsilon}_t^{(k)}$ model spatial and temporal correlation respectively. The values of the index *k* correspond to the sporozoite models for *An. funestus* (*k* = 1), *An. gambiae* (*k* = 2) and mosquito density models for *An. funestus* (*k* = 3) and *An. gambiae* (*k* = 4) respectively. We assume a stationary Gaussian spatial process that is, spatial correlation is considered to be a function of distance only and not of the locations themselves an autoregressive process of order assuming that temporal correlation is present only between successive time points to capture temporal correlation. Seasonal trends *f*_(*k*)_(*t*) are modeled via a trigonometric function with a period *T* = 12 months, ${f}_{(k)}(t)={a}_{1k} \cos \left(\frac{2\pi }{T}t\right)+{a}_{2k} \sin \left(\frac{2\pi }{T}t\right),t=1,\dots, 12$. The peak months of the wet and dry season are calculated by *t*_*k*_ = arctan(*a*_1*k*_/*a*_2*k*_) × (*T*/2*π*) and (*t*_*k*_ + *T*/2), respectively [13].

### Determining important predictors and lag times using variable selection

Bayesian variable selection was carried out to determine the most important climatic factors including distance to water bodies and lag variables for each climatic factor (NDVI, day LST, night LST, Rainfall) using a variable selection approach known as stochastic search [14]. In particular, for each predictor ${\underset{\sim }{X}}_p$ we introduce a binary indicator parameter *I*_*p*_ suggesting presence (*I*_*p*_ = 1) or absence (*I*_*p*_ = 0) of the predictor from the model. Furthermore, we assume a mixture prior for the corresponding regression coefficient *β*_*p*_ that is ${\beta}_p\sim \left(1-{I}_p\right)N\left(0,{\upsilon}_0{\tau}_p^2\right)+{I}_pN\left(0,{\tau}_p^2\right)$ proposing a non-informative prior for *β*_*p*_ in case ${\underset{\sim }{X}}_p$ is included in the model and an informative normal prior with a variance close to zero (i.e. *υ*_0_ = 10^− 3^) shrinking *β*_*p*_ to zero if ${\underset{\sim }{X}}_p$ is excluded from the model. A Bernoulli prior is assumed for the indicator, *I*_*p*_ ~ *Be*(0.5). For climatic variables with lag effects we introduce a multivariate binary indicator with categories corresponding to the six lag periods and an additional category to allow exclusion of that variable. In this case we consider a multinomial prior for the indicator with equal probabilities among the categories. Variable selection was applied on the climatic factors included in both parts of the models, i.e. the mixing proportion of zeros and the mean parameters of the data distributions. The predictors that are identified as important are those with posterior inclusion probability greater than or equal to 50% [15].

Model fit was carried out using Markov chain Monte Carlo simulation. We ran a two-chain Gibbs sampler over 100,000 iterations using a burn-in of 5000 iterations. Convergence was assessed by the Gelman and Rubin diagnostic [16] and kernel density plots using the coda routine in R.

Details on remaining prior distribution and implementation are given in the Additional file 1: Figure S1. The analysis was carried out in OpenBUGS version 3.2.3 (Imperial College and Medical Research Council, London, UK).

### Entomological inoculation rate (EIR)

Bayesian kriging [17] was used to predict the mosquito density and the number of infected mosquitoes for each species over a regular grid of 33 605 pixels at 250 × 250 m^2^ spatial resolution covering the study area. The entomological inoculation rate (EIR) is defined as the product of human biting rate and the sporozoite rate. The human biting rate is the mean number of bites received per host and per night. It is approximated by mosquitoes captured using human landing catches (host-seeking mosquitoes). We used a conversion factor of 1.605 [18] to transform light trap catches densities into human landing catches. The light trap density was calculated by dividing the number of mosquitoes caught using the CDC light traps by the number of trap-nights. At a specific pixel *i* and month *t,* a sample of size 1,000 was drawn from the predicted posterior distribution of the species-specific mosquito densities using the zero-inflated negative binomial data distribution. In addition, a sample of the number of infected mosquitoes (from each species) was simulated from the predictive posterior distribution of sporozoite rates based on the zero-inflated binomial with a binomial count equal to the predicted mosquito density. The overall EIR estimate at a given location and month is based on the sample-based mean number of infected mosquitoes (from both species) multiplied by the conversion factor. Bayesian kriging was done in Fortran 95 (Digital Equipment Corporation) using standards numerical libraries (Numerical Algorithms Group Ltd.).

### Model validation

Model fit was carried out on a randomly selected locations subset (85%) of the dataset (training set). The remaining 15% was used for model validation (testing test). These subsets were selected by assigning a uniform distribution on the locations. The predictive performance of the models was assessed by calculating the proportion of test locations with the outcome variable included in the credible intervals (CI) with varying probability coverage ranging from 5% to 95% of the posterior predictive distribution and the mean square error between the observed and predicted data [19].

## Results

### Vectorial density

**Figure 1:**
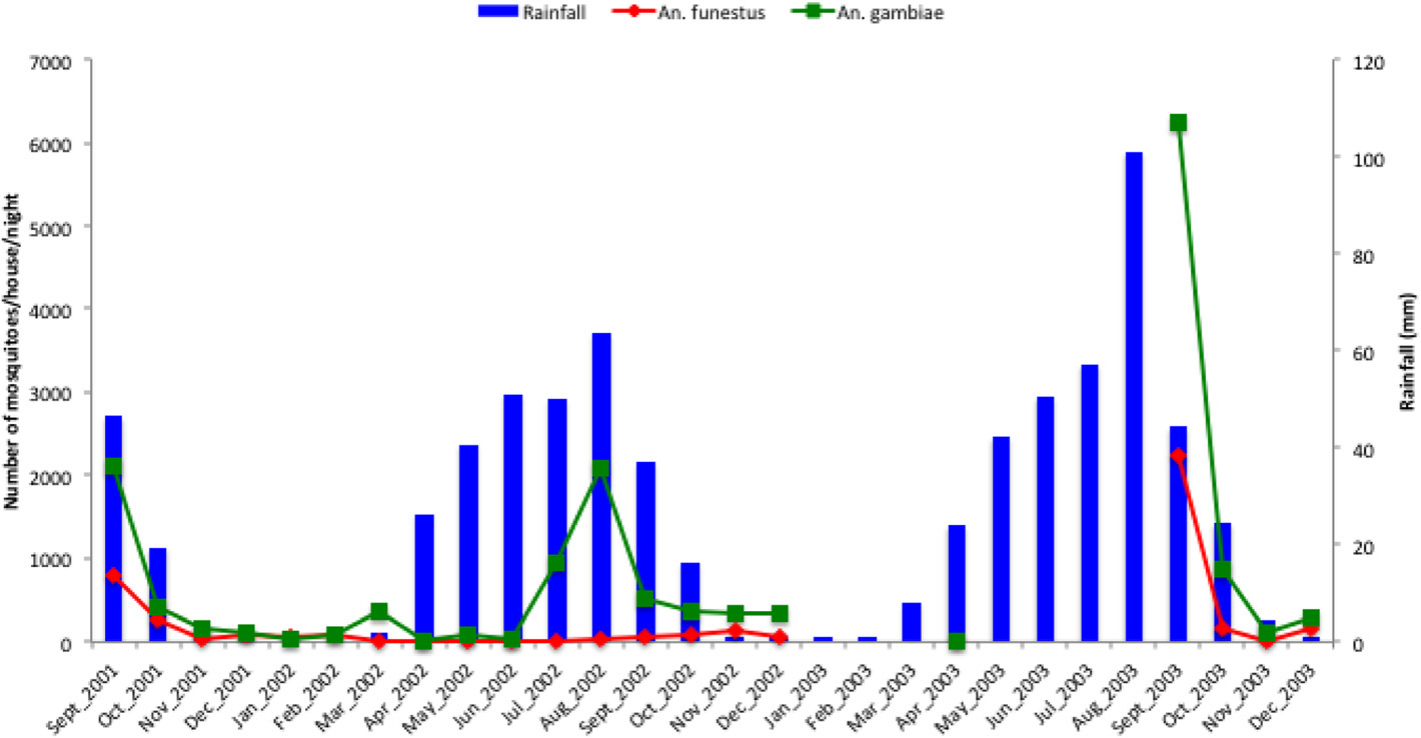
**The distributions of the species-specific densities and rainfall throughout the study period.**

A total of 13,132 anopheline mosquitoes were trapped in 550 unique locations over the study period. *An.°gambiae* was the predominant vector species representing 79% of the total Anopheles mosquitoes collected. The remaining 21% consisted of *An. funestus*. About 35% of the survey locations had no *An. gambiae* mosquitoes. This percentage reaches 41% for *An. funestus*. The peak collecting period for *An. gambiae* species coincided with the peak of the rainy season (August) while for *An. funestus* it was in September. However the density of *An. funestus* remained comparatively low throughout the study period. The distributions of the species-specific densities and rainfall throughout the study period are given in Figure 1.

### Mosquito infectivity

**Figure 2:**
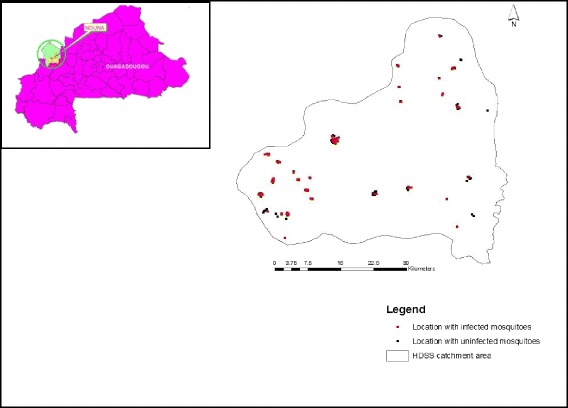
**Geographical locations (top left) and surveyed locations with infected and uninfected mosquitoes.**

**Figure 3:**
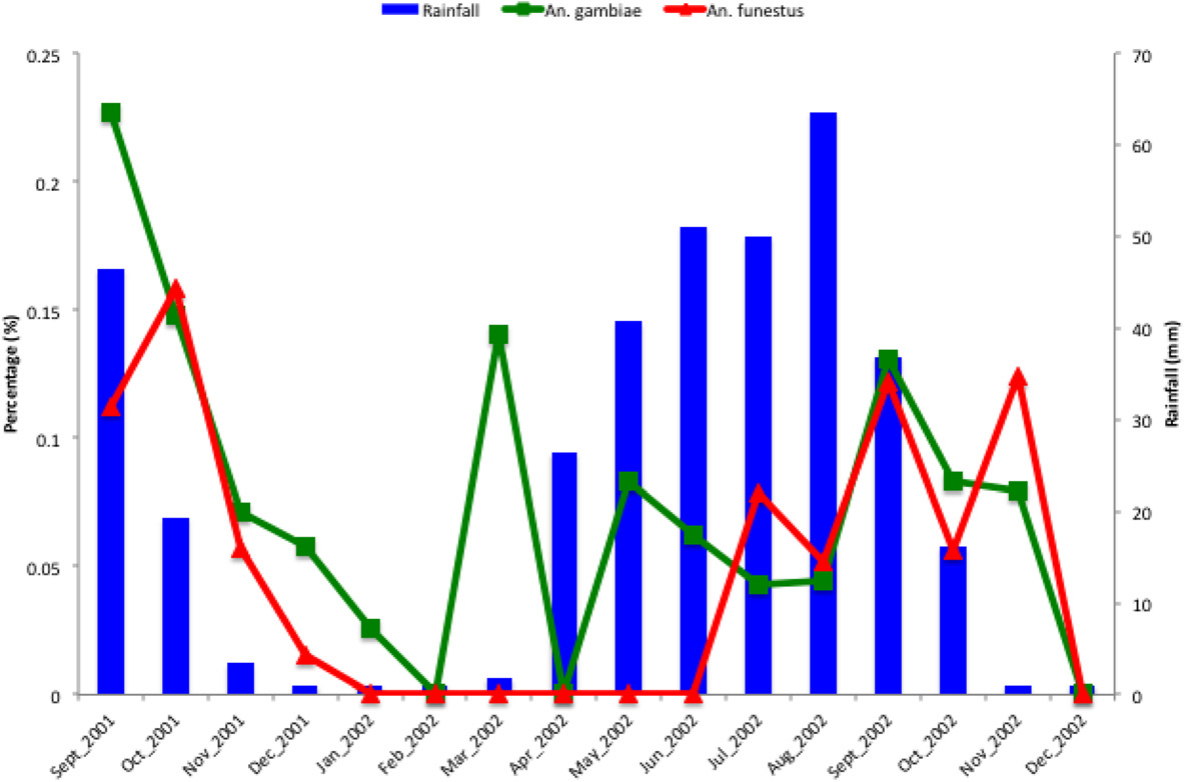
**Monthly patterns of species-specific infectivity and rainfall.**

A total of 5,668 mosquitoes were tested for the presence of circumsporozoite antigens out of which 4,230 (74.64%) were *An. gambiae* species. The overall sporozoite rate (SR) was 7.64% (95% CI 7.63-7.65). *Plasmodium falciparum* infection was detected in 9.24% of *An. gambiae* species and 2.92% of *An. funestus* species. Sporozoite rates tend to be higher in the Western part of the study area. Figure 2 presents the geographical location of the study area (top left) and surveyed locations with infected and uninfected mosquitoes. The monthly patterns of infected mosquitos and rainfall by species are depicted in Figure 3.

### Variable selection

Results of the variable selection in Table 2 indicate that rainfall and vegetation during the current month (Lag0), and day temperature during the month preceding the collection (Lag1) are important predictors of *An. funestus* density. The rise of *An. gambiae* density depends on suitable climatic conditions over a longer period of time such as rainfall during the current and previous two months and night temperatures during the previous two months. The proportion of “structural” or “excess” zeros is influenced by day and night temperatures for both species. However, for *An. gambiae* densities, the mixing proportion is associated with a longer lag time, that is second month (Lag2) and two previous and current months (Lag5) for day and night temperatures, respectively. Furthermore, distance to water bodies appears to be an important predictor of the mixing proportion of zeros for *An. funestus* densities.

**Table 2:** **Lag times and predictors selected by the variable selection**

**Model**	**Zero-inflated binomial**	**Zero-inflated binomial**	**Zero-inflated negative binomial**	**Zero-inflated negative binomial**
**Binomial component**	***An. funestus***	***An. gambiae***	***An. funestus***	***An. gambiae***
**Parameter**				
Rainfall	Lag 3	Lag 0	Lag 0	Lag 5
Vegetation (NDVI)	-	-	Lag 0	-
Day temp (LSTD)	Lag 4	Lag 3	Lag 1	-
Night temp (LSTN)	-	Lag 5	-	Lag 4
Distance to water body	Yes	No	No	No
**Mixing Proportion**				
Rainfall	-	-	-	-
Vegetation (NDVI)	-	-	-	-
Day temp (LSTD)	-	-	Lag 1	Lag 2
Night temp (LSTN)	-	-	Lag 1	Lag 5
Distance to water body	Yes	Yes	Yes	No

**Legend:**

Lag 0: Average over the current month.

Lag 1: Average of the environmental covariate over the previous month.

Lag 2: Average of the environmental covariate over second previous month.

Lag 3: Average of the environmental covariate over the current and the previous month.

Lag 4: Average of the environmental covariate over the previous and the second previous month.

Lag 5: Average of the environmental covariate over the current, previous and the second previous month.

The most important climatic predictors of the sporozoite rates of *An. funestus* are rainfall during the current and previous month (Lag3) and day temperature during the two previous months (Lag4). Rainfall of the month of collection (Lag0) and more distant lag times for day (Lag3 corresponding to the current and previous month) and night temperatures (Lag5, i.e. current and the two previous months) appear to be the most important predictors of *An. gambiae* sporozoite rates. Distance to water body is the only important predictor of the proportion of “excess” zeros in the sporozoite rates for both species.

### Model-based vectorial density

Positive effects of rainfall and vegetation during the current month showed important associations with the density of *An. funestus*. The distance at which the spatial correlation is less than 5% is equal to 10 km (95% Bayesian credible interval (BCI): 3-83 km). The phase of 0.65 radials suggested that the peak of the *An. funestus* density occurs in the month of September and the minimum in the month of March. The effect of predictors associated to the mixing proportion of the zero-inflated distribution was not important. The probability of the excess zeros is highest in the month of November and the lowest in the month of April.

**Figure 4:**
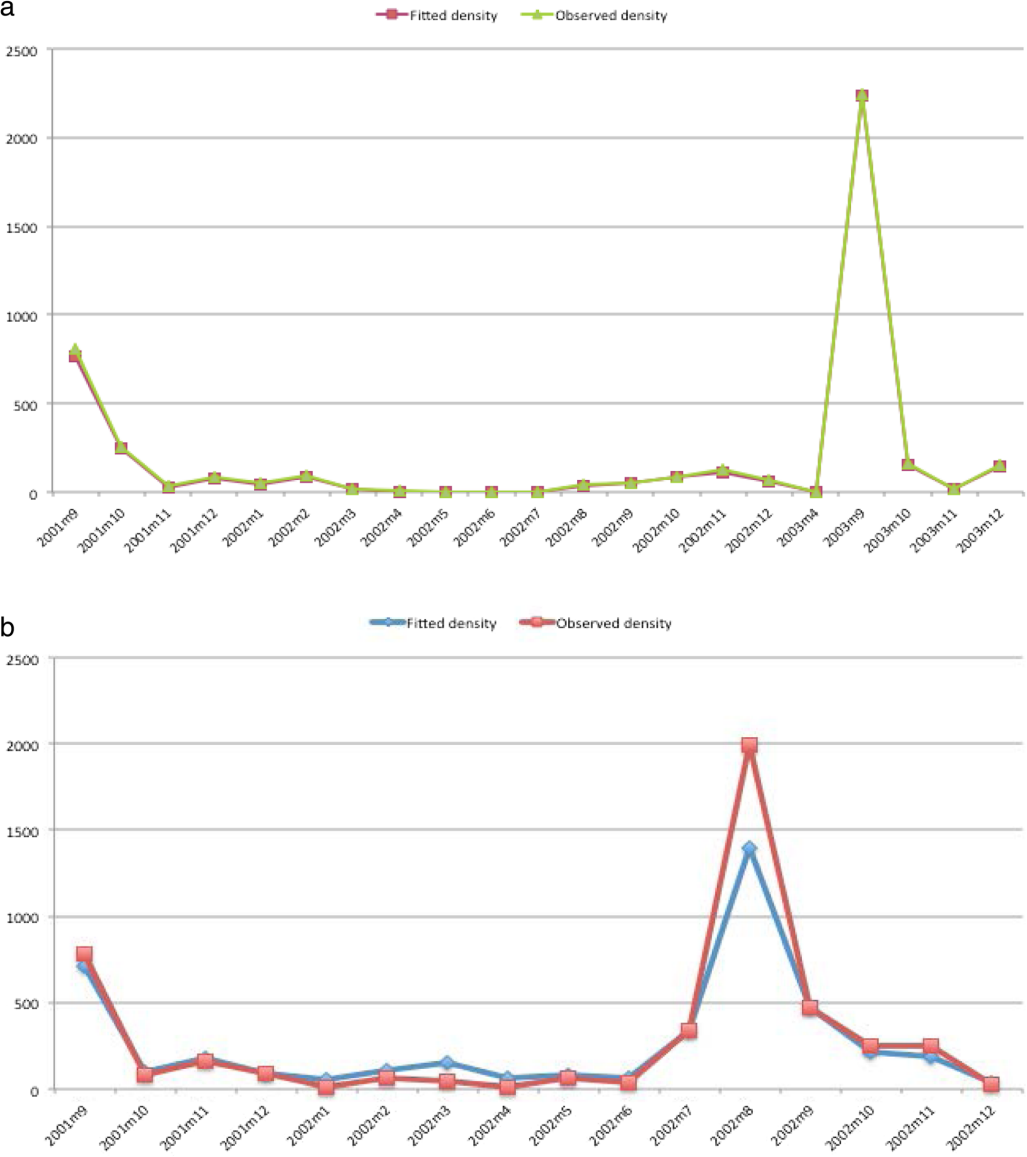
**Monthly patterns of observed and fitted indoor residual densities. (a)** Averaged over spatial locations of *An. funestus.* **(b)** Averaged over spatial locations of *An. gambiae.*

Rainfall (during the current and two previous months) and night temperature (during the two previous months) are important predictors, negatively associated with *An. gambiae* density. Spatial correlation is not important (<0.05) beyond 5 km (95% BCI: 1-35 km). The temporal and the spatial variations are respectively 0.76 (95% BCI: 0.48-1.26) and 0.54 (95% BCI: 0.22-1.13). The maximum *An. gambiae* density occurs in the month of August and the minimum in the month of January. The probability of excess is related positively with the day temperature during the two previous months and negatively with the night temperature during the current and two previous months. The maximum probability of excess zero occurs in November and the minimum in April. Table 3 presents the posterior estimates of the ZINB model for both species. Figure 4 shows the monthly pattern of observed and fitted density (averaged over spatial locations) respectively for *An. funestus* and *An. gambiae.*

**Table 3:** **Posterior estimates obtained from the geostatistical zero-inflated negative binomial (ZINB) models**

**Parameters**	***An. funestus***	***An. gambiae***
	**Median (95% BCI)**	**Median (95% BCI)**
Intercept	-0.38 (-1.09, 0.46)	1.85 (-0.11, 3.56)
Year2	0.24 (-0.64, 1.07)	-1.01 (-2.75, 0.39)
Rainfall	0.87 (0.021, 1.71)	-2.33 (-4.67, -0.2)
Vegetation (NDVI)	1.12 (0.63, 1.65)	-
Day temp (LSTD)	-0.78 (-1.56, 0.00)	-
Night temp (LSTN)	-	-1.3 (-1.9, -0.64)
Amplitude	3.53 (3.50, 3.56)	5.88 (5.83, 5.93)
Shift/phase	0.648 (0.645, 0.653)	-1.159 (-1.163, -1.155)
Dispersion (r)	0.45 (0.32, 0.63)	0.93 (0.71, 1.24)
Spatial variation	0.90 (0.33, 2.03)	0.54 (0.22, 1.13)
Range (km)^a^	1 (3, 83)	5 (1, 35)
Temporal variation	-	0.76 (0.48, 1.26)
**Parameters**	**Mixing proportion**	
	***An. funestus***	***An. gambiae***
	**Median (95% BCI)**	**Median (95% BCI)**
Intercept	-14.42 (-27.08, -3.57)	-11.06 (-18.80, -3.47)
Year2	0.24 (-0.64, 1.07)	4.42 (-5.67, 11.66)
Distance to water body	-3.52 (-12.43, 3.51)	-
Rainfall	-	-
Vegetation (NDVI)	-	-
Day temp (LSTD)	4.14 (-9.38, 21.23)	3.21 (0.89, 5.89)
Night temp (LSTN)	5.13 (-5.08, 14.83)	-1.99 (-5.57, -0.57)
Amplitude	21.92 (21.69, 22.16)	4.03 (3.96, 4.18)
Shift/phase	1.06 (1.05, 1.07)	2.25 (2.22, 2.27)

^a^minimum distance in kilometer at which the spatial correlation remains important, BCI = Bayesian Credible Interval.

### Model-based mosquito infectivity

Table 4 presents the posterior estimates of the ZIB model for both species. The effects of predictors appeared not to be important for both species. The maximum of *An. funestus* infectivity occurs in the month of August and the minimum in April. Estimates of the seasonality parameters in the mixing proportion indicate that the probability of “structural” zeros is maximum in October and minimum in the month of April.

**Table 4:** **Posterior estimates obtained from the geostatistical zero-inflated binomial (ZIB) models**

**Parameters**	***An. funestus***	***An. gambiae***
	**Median (95% CI)**	**Median (95% CI)**
Intercept	-6.65 (-14.8, -1.42)	-1.82 (-4.21, 4.14)
Year2	-0.67 (-3.36, 2.10)	-0.92 (-4.32, 1.49)
Distance to water body	0.10 (-1.54, 1.83)	-
Rainfall	-1.42 (-4.84, 1.51)	-0.16 (-2.06, 1.94)
Vegetation (NDVI)	-	-
Day temp (LSTD)	-0.25 (-3.53, 2.64)	-0.47 (-1.13, 0.08)
Night temp (LSTN)	-	0.05 (-0.72, 0.81)
Amplitude	7.25 (7.12, 7.38)	2.50 (2.45, 2.54)
Shift/phase	-1.89 (-1.94, -1.85)	2.72 (2.68, 2.75)
Spatial variation	0.67 (0.22, 2.78)	0.51 (0.22, 1.19)
Range (km)^a^	0.05 (0.01, 0.44)	0.05 (0.01, 0.27)
Temporal variation	-	0.99 (0.53, 2.26)
**Parameters**	**Mixing proportion**	
	***An. funestus***	***An. gambiae***
	**Median (95% CI)**	**Median (95% CI)**
Intercept	-2.96 (-20.12, 11.79)	-11.45 (-26.0, -0.0015)
Year2	1.88 (-17.24, 15.2)	-3.25 (-18.15, 9.07)
Distance to water body	11.5 (-7.67, 27.29)	3.546 (-9.625, 11.39)
Rainfall	-	-
Vegetation (NDVI)	-	-
Day temp (LSTD)	-	-
Night temp (LSTN)	-	-
Amplitude	11.09 (10.88, 11.30)	4.03 (3.96, 4.10)
Shift/phase	1.19 (1.15, 1.23)	2.25 (2.22, 2.27)

^a^minimum distance in kilometer at which the spatial correlation is significant at 5%, CI = credible interval.

Sporozoite rates in *An. gambiae* take the largest and lowest values in November and April, respectively.

**Figure 5:**
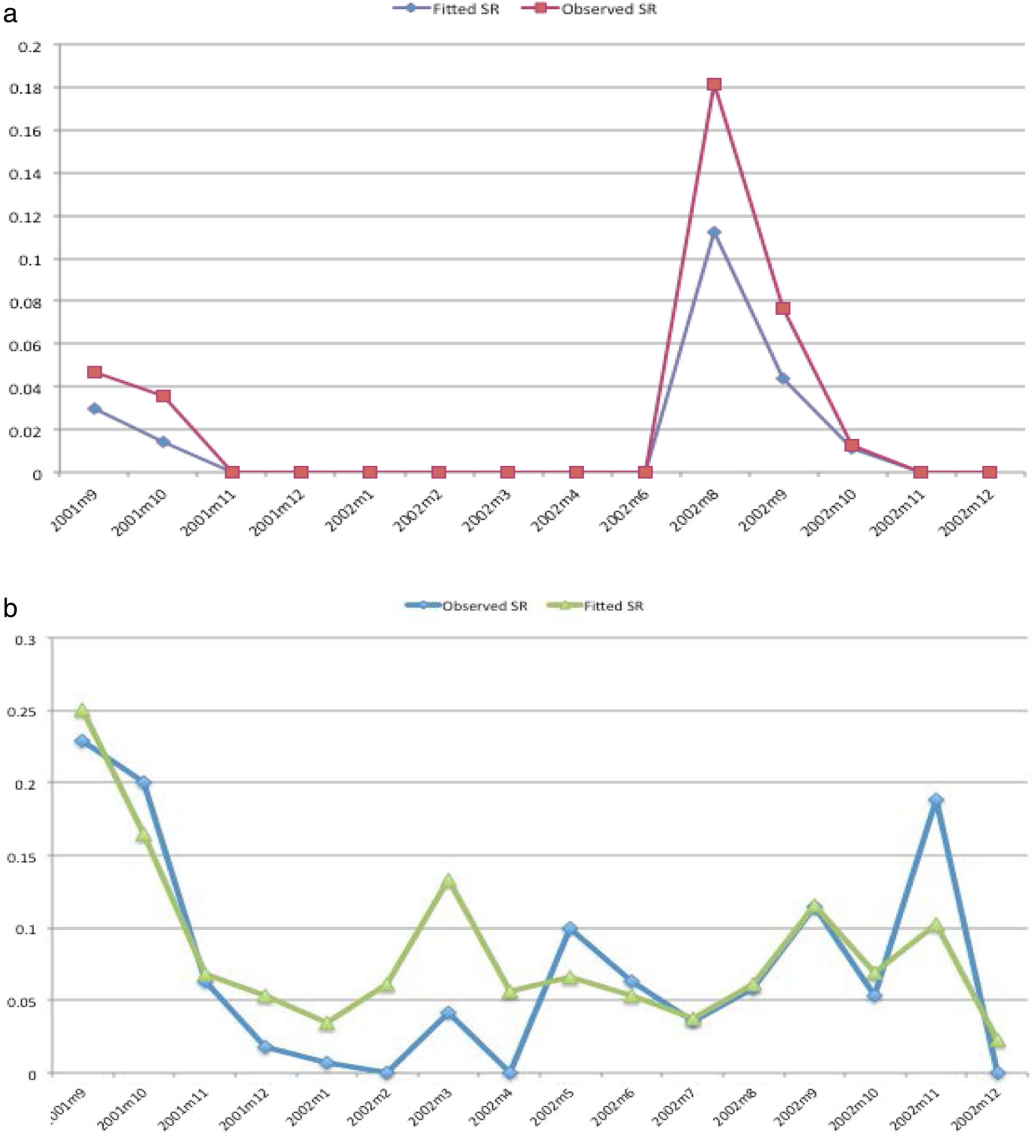
**Monthly pattern of observed and fitted sporozoite rate. (a)** Averaged over spatial locations of *An. funestus.* **(b)** Averaged over spatial locations of *An. gambiae.*

The probability of “structural” zeros is higher in December and lower in June. Variation in time is larger than the one in space. Furthermore, the distance at which the correlation coefficient falls below 5% (spatial correlation) is deemed unimportant for both *An. funestus* and *An. gambiae* (95% BCI: 1-44 km and 1-27 km, respectively). Figure 5a-b shows the monthly pattern of observed and fitted sporozoite rate (averaged over spatial locations) respectively for *An. funestus* and *An. gambiae.*

### Entomological inoculation rate (EIR)

**Figure 6:**
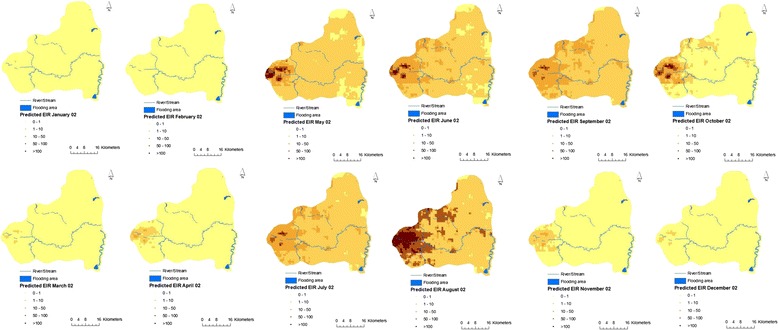
**Monthly EIR estimates of the median predictive posterior distribution at 250 by 250 m** ^**2**^ **resolutions.**

The annual EIR averaged across the area was 131.4 infective bites per person for 2002. Figure 6 depicts monthly EIR estimates of the median predictive posterior distribution at 250 by 250 m^2^ resolutions within the HDSS site. The high transmission season is during May–October; however there are some “high-transmission” areas in the western part during November. In fact, the western region has the highest EIR estimates across the whole HDSS catchment area.

### Model validation

Model validation showed that 92% and 73% of the test locations had sporozoite rate falling within the 95% credible interval estimated from the zero inflated spatial binomial model and zero inflated spatio-temporal model respectively for *An. funestus* and *An. gambiae*.

**Figure 7:**
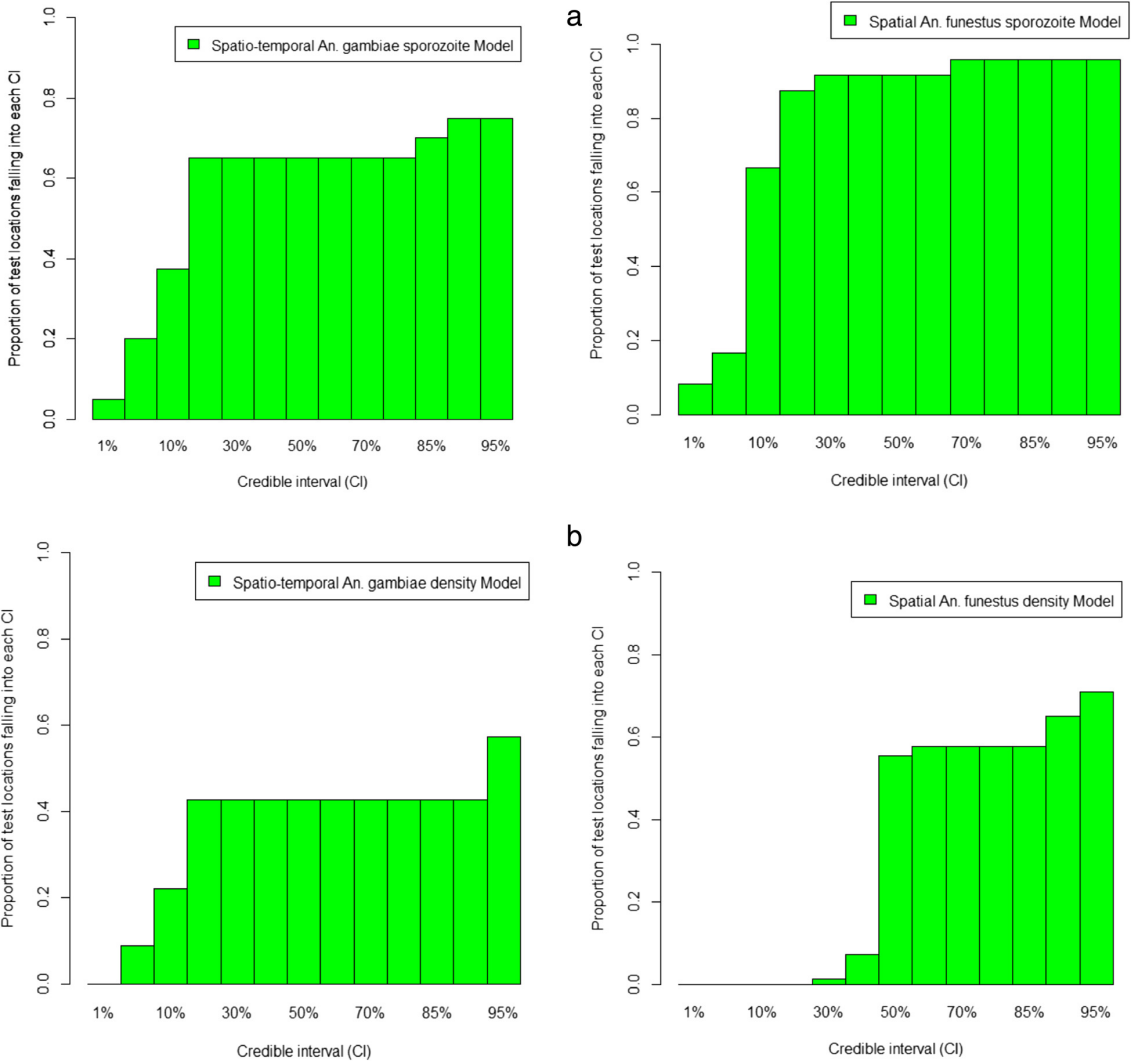
**Proportions of test locations falling in between 5% and 95% credible intervals. (a)** Proportions of test locations with sporozoite rate falling in between 5% and 95% credible intervals. **(b)** Proportions of test locations with mosquito density falling in between 5% and 95% credible intervals.

Density models validation showed that 73% and 58% of the test locations had mosquito densities falling within the 95% credible interval estimated from the zero inflated spatial negative binomial model and zero inflated spatio-temporal negative binomial model respectively for *An. funestus* and *An. gambiae* density models. However the zero inflated spatio-temporal models included higher proportion of test locations in the lowest credible intervals compared to the zero inflated spatial negative binomial. Figure 7a-b shows the proportions of test locations with respectively sporozoite rate and mosquito density falling in between 5% and 95% credible intervals of the posterior predictive distribution.

## Discussion

In this study we described and quantified malaria transmission heterogeneity in the Nouna HDSS, using a comprehensive entomological dataset and rigorous spatio-temporal models, which include Bayesian variable selection and take into account zero-inflation. The models determine the elapsing time between climate suitability and malaria transmission and estimate spatio-temporal patterns of transmission. Malaria transmission is mainly driven by two efficient vectors namely *Anopheles gambiae* and *An. funestus*, which co-exist geographically across the study area. The transmission fluctuated over the study period indicating seasonal, spatial and temporal variation within such a small geographic extend (1775 km^2^). These findings corroborate previous studies concluding a very heterogeneous malaria distribution which is prone to great variations between villages and compounds [2,3,6]. Transmission intensity measured by EIR in Nouna HDSS was high (>100 ib/p/y) especially in the rainy season. A seasonal pattern was observed in mosquito densities and in sporozoite rates for both species. The high transmission season starts from May throughout October with the peak transmission occurring in September.

The negative relationship between night temperatures and *An. gambiae* mosquito density in our results possibly imply that although the high temperatures of the study area (average daily minimum: 20-28.1°C, maximum: 29.5-37.2°C) are suitable for stable malaria transmission [20], a spell of relief from the heat mainly in the night is also a key determinant for mosquito development and survival.

Rainfall is associated with the densities of both species; however the direction of the effect is different. A negative association with *An. gambiae* density may suggest that although rainfall remains an important factor for the development of this species, consecutive heavy rainfall (over the current and the two previous months as shown by the Lag5 effect) may flush away all suitable *An. gambiae* breeding sites, therefore *An. gambiae* is a rainy-dependant species which favours temporary and shallow breeding sites. A positive association with *An. funestus* density indicates that rainfall is important for the development and survival of this species which predominantly develops in permanent water bodies with emerging vegetation [21]. This result is consistent with the positive important association between NDVI (a proxy measure of vegetation) and *An. funestus* density and the lack of association between NDVI with *An. gambiae* density (NDVI was not identified as a potential predictor of *An. gambiae* density).

The shortest distance at which the spatial correlation was below 5% for sporozoite rate was 5 km for both species. However, for the density it is twice as much for *An. funestus* than that for *An.* Gambiae in spite of wide credible intervals associated with both estimates. The negative association between rainfall and sporozoite rate (although not significant) for both species can be explained by the sporogony cycle in relation to mosquito survival. Furthermore, only adult mosquitoes that have successfully taken a blood meal carry sporozoites while many young newly emerged mosquitoes shortly after the onset of the rainy season may reduce the proportion of older ones in the population. Similar results were also found by [22] and [23].

The lack of association between distance to water bodies especially *An. gambiae* may be explained by the fact the water bodies considered are mostly large permanent and semi-permanent ones and does not include small breeding sites which are favoured by *An. gambiae*. The zero-inflated (ZI) model formulations that were adopted in our study allow us to account for the structural zeros that may arise due to some factors that have not been considered in the study. For example, vector control interventions targeting adult mosquitoes that are likely to be infective or proximity to temporary water bodies where it is likely to find many young newly emerged (not yet infective) mosquitoes.

Interestingly, high EIR estimates are observed in the western part of the study area, which is located to a large extent in shallows that are extensively used by local populations for rice cultivation. The transmission in this area remained high even during the dry season. Figure 3 in the Additional file 1: Figure S1 shows the monthly patterns of observed and fitted of sporozoite rate (a,b)-(c,d) and densities (e,f)-(g,h) averaged over spatial locations respectively in high and low EIR regions of the study area and for *An. funestus* and *An. gambiae*. The observed and fitted sporozoite rate and densities plots display similar patterns in both the low and high EIR regions.

The EIR maps clearly depict a strong spatial and temporal heterogeneity despite the relatively small geographical extent of the study area.

These maps are valuable tools in identifying malaria "high-transmission" areas and in prioritizing timely, control interventions. The high spatial resolution EIR estimates are also important in addressing research questions such as the relationship between malaria transmission intensity and mortality.

The lag time analysis indicated short elapsing periods between climatic suitability and rise of *An. funestus* densities as opposed to longer times required by *An. gambiae*. A possible explanation could be that rainfall quickly dries out or it streams into shallows (in case of heavy rainfall) where water is collected for an extended period thus favouring the development of *An. funestus.* Suitable breeding sites for *An. gambiae* appear only after successive rainfalls that lead to soil saturation. Understanding the lag times between climate suitability and change in malaria transmission is important not only for delivering interventions at the right time but also for developing predictive models to support early warning systems (EWS). In many studies the choice of environmental predictors is based on biological plausibility rather than assessing whether plausibility is supported by the data generated by the study site. However, local conditions influence transmission patterns, therefore rigorous modelling approaches that take into account and estimate lag times in climatic factors are needed to increase model predictive ability.

In this study we used and systematically examined different lag structures through Bayesian variable selection implemented within a geostatistical model. Modeling lag effects via distributed lag models [24-26] is an alternative approach to the one used in this paper, however this approach assumes that the different climatic proxies are available on a daily scale or aggregated over a common temporal resolution (e.g. month). We are currently comparing both approaches.

To our knowledge, this is the first effort in estimating and comparing the lag time between climatic suitability and malaria transmission between the two vector species. The results improve our understanding of the dynamics of malaria transmission. However it is worth noticing that the associations found in this study area may not necessarily apply in different eco-climatic zones and further work in the area could clarify how lag effects depend on ecological zone. Model based estimates of transmission can identify high transmission areas in order to prioritise interventions and support research in malaria epidemiology.

## Conclusions

This study identified the most important environmental/climatic variables associated with malaria transmission in the Nouna region in Burkina Faso and determined the lag times between climate suitability and change in entomological indices. The prediction maps of entomological inoculation rate (EIR) surfaces depict a strong spatial and temporal heterogeneity. Our results contribute to a better understanding of the interplay between environmental/climatic conditions and malaria transmission, which is important not only for delivering interventions at the right time but also for developing predictive models to support early warning systems (EWS).

## Appendix

### Prior distributions and model implementation

We assume that *ϕ*_*i*_^(*k*)^’s latent observations from a stationary Gaussian spatial process $N\left(0,{\sigma}_{1k}^2{R}^{(k)}\right)$. *R*^(*k*)^ models spatial correlation as an exponential function of distance *d*_*ij*_ between any pairs of locations *i* and *j* that is ${R}_{ij}^{(k)}= \exp \left({d}_{ij};{\rho}_k\right)$ and *ρ*_*k*_ is a measure of the rate of the correlation decay with distance. The value 3/*ρ*_*k*_ estimates the maximum distance at which the spatial correlation is significant at 5% [27]. The ${\sigma}_{1k}^2$ measures the within-location variation. Temporal correlation was introduced by monthly random effects and modelled by autoregressive (AR) process of order 1 that is ${\varepsilon}_t^{(k)}\sim N\left({\theta}^{(k)}{\varepsilon}_{t-1}^{(k)},{\sigma}_{2k}^2\right)$ and ${\varepsilon}_t^{(k)}\sim N\left(0,{\sigma}_{2k}^2/1-{\theta}_k\right)$. ${\sigma}_{2k}^2$ and *θ*_*k*_ are the temporal variance and autocorrelation parameters respectively.

For the regression coefficients we adopt a non-informative normal prior distribution with large variance. We further assumed a normal prior distributions with mean zero and large variance for the coefficients of the seasonal trends, that is *a*_1*k*_ and *a*_2*k*_ ~ *N*(0, 10^2^). For the spatial parameters ${\sigma}_k^2$ and *ρ*_*k*_ we adopt inverse gamma and gamma prior distributions respectively, that is ${\sigma}_k^2\sim IG\left(2.01,1.01\right)$ and *ρ*_*k*_ ~ *G*(0.1, 0.1). Covariates were standardized in order to acquire better correlation properties and reduce the Markov chain Monte Carlo simulation computational time [28].

Convergence was assessed by Gelman and Rubin diagnostic [16] and kernel density plots.

Exploratory analysis was carried out in STATA 11 (Stata Corporation, College Station, Texas, USA).

## Additional file

Additional file 1: Figure S1. (a): Monthly pattern of observed and fitted sporozoite rate of *An. funestus:* averaged over spatial locations in western (high EIR) region of the study area. (b): Monthly pattern of observed and fitted sporozoite rate of *An. funestus:* averaged over spatial locations in eastern (low EIR) region of the study area. (c): Monthly pattern of observed and fitted densities of *An. gambiae:* averaged over spatial locations in western (high EIR) region of the study area. (d): Monthly pattern of observed and fitted densities of *An. gambiae:* averaged over spatial locations in western (low EIR) region of the study area. (e): Monthly pattern of observed and fitted densities of *An. funestus:* averaged over spatial locations in western (high EIR) region of the study area. (f): Monthly pattern of observed and fitted densities of *An. funestus:* averaged over spatial locations in western (low EIR) region of the study area. (g): Monthly pattern of observed and fitted densities of *An. gambiae:* averaged over spatial locations in western (high EIR) region of the study area. (h): Monthly pattern of observed and fitted densities of *An. gambiae:* averaged over spatial locations in western (low EIR) region of the study area.
