# Supplementary material for: Bayesian variable selection in modelling geographical heterogeneity in malaria transmission from sparse data: an application to Nouna Health and Demographic Surveillance System (HDSS) data, Burkina Faso
Source: Parasit Vectors. 2015 Feb 22;8:118. doi: 10.1186/s13071-015-0679-7 (PMC4365550; doi:10.1186/s13071-015-0679-7)

**Additional file 1: Figure S1.** (a): Monthly pattern of observed and fitted sporozoite rate of *An. funestus:* averaged over spatial locations in western (high EIR) region of the study area. (b): Monthly pattern of observed and fitted sporozoite rate of *An. funestus:* averaged over spatial locations in eastern (low EIR) region of the study area. **(c**): Monthly pattern of observed and fitted densities of *An. gambiae:* averaged over spatial locations in western (high EIR) region of the study area. (d): Monthly pattern of observed and fitted densities of *An. gambiae:* averaged over spatial locations in western (low EIR) region of the study area. (e): Monthly pattern of observed and fitted densities of *An. funestus:* averaged over spatial locations in western (high EIR) region of the study area. (f): Monthly pattern of observed and fitted densities of *An. funestus:* averaged over spatial locations in western (low EIR) region of the study area. (g): Monthly pattern of observed and fitted densities of *An. gambiae:* averaged over spatial locations in western (high EIR) region of the study area. (h): Monthly pattern of observed and fitted densities of *An. gambiae:* averaged over spatial locations in western (low EIR) region of the study area.


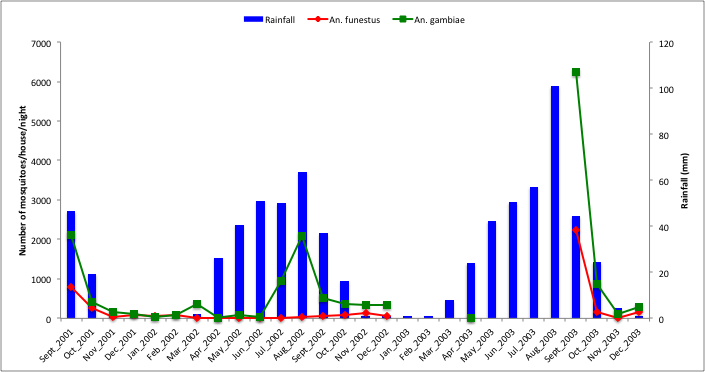

Supplement: Additional file 1: Figure S1. — (a): Monthly pattern of observed and fitted sporozoite rate of An. funestus: averaged over spatial locations in western (high EIR) region of the study area. (b): Monthly pattern of observed and fitted sporozoite rate of An. funestus: averaged over spatial locations in eastern (low EIR) region of the study area. (c): Monthly pattern of observed and fitted densities of An. gambiae: averaged over spatial locations in western (high EIR) region of the study area. (d): Monthly pattern of observed and fitted densities of An. gambiae: averaged over spatial locations in western (low EIR) region of the study area. (e): Monthly pattern of observed and fitted densities of An. funestus: averaged over spatial locations in western (high EIR) region of the study area. (f): Monthly pattern of observed and fitted densities of An. funestus: averaged over spatial locations in western (low EIR) region of the study area. (g): Monthly pattern of observed and fitted densities of An. gambiae: averaged over spatial locations in western (high EIR) region of the study area. (h): Monthly pattern of observed and fitted densities of An. gambiae: averaged over spatial locations in western (low EIR) region of the study area. [file 13071_2015_679_MOESM1_ESM.doc]
